# Phenotyping of Leukocytes and Leukocyte-Derived Extracellular Vesicles

**DOI:** 10.1155/2016/6391264

**Published:** 2016-04-18

**Authors:** Lotte Hatting Pugholm, Rikke Bæk, Evo Kristina Lindersson Søndergaard, Anne Louise Schacht Revenfeld, Malene Møller Jørgensen, Kim Varming

**Affiliations:** Department of Clinical Immunology, Aalborg University Hospital, 9000 Aalborg, Denmark

## Abstract

Extracellular vesicles (EVs) have a demonstrated involvement in modulating the immune system. It has been proposed that EVs could be used as biomarkers for detection of inflammatory and immunological disorders. Consequently, it is of great interest to investigate EVs in more detail with focus on immunological markers. In this study, five major leukocyte subpopulations and the corresponding leukocyte-derived EVs were phenotyped with focus on selected immunological lineage-specific markers and selected vesicle-related markers. The leukocyte-derived EVs displayed phenotypic differences in the 34 markers investigated. The majority of the lineage-specific markers used for identification of the parent cell types could not be detected on EVs released from monocultures of the associated cell types. In contrast, the vesicular presentation of CD9, CD63, and CD81 correlated to the cell surface expression of these markers, however, with few exceptions. Furthermore, the cellular expression of CD9, CD63, and CD81 varied between leukocytes present in whole blood and cultured leukocytes. In summary, these data demonstrate that the cellular and vesicular presentation of selected lineage-specific and vesicle-related markers may differ, supporting the accumulating observations that sorting of molecular cargo into EVs is tightly controlled.

## 1. Introduction

Extracellular vesicles (EVs) are a heterogeneous group of vesicles that can be subdivided based on their size, biogenesis, and molecular composition. Using the biogenesis as a classification tool, EVs can be divided into three major groups, namely, exosomes, microvesicles (MVs), and apoptotic bodies [1–3]. Even though the molecular composition of these three subsets of EVs is different, several markers overlap. So far, identification of a specific marker that with certainty can distinguish or identify the particular EV subset still awaits [4]. It can be expected that the different EV subsets may cover different biological roles, but the function of EVs has also been described to depend both on the cellular source and on the recipient tissue/cell [2]. Nevertheless, it is now recognized that EVs are involved in numerous physiological processes, including intercellular communication and delivery of proteins, lipids, and genetic material to recipient cells [2, 3, 5–7]. In addition, EVs have been associated with the development and progression of different pathological conditions, including cancer and infectious diseases [8–15].

The immunological effects of EVs comprise a broad range of mechanisms, including immune activation, immune suppression, and modulation of immune surveillance. Cells from both the innate and the adaptive immune system have been shown to release EVs, such as T and B cells, dendritic cells (DCs), macrophages, mast cells, and natural killer (NK) cells [16–24]. The effect of EVs is directly related to their molecular composition and several studies have ascribed an immunostimulatory effect of EVs to the presence of a very specific molecular content [20, 23, 25–31]; for instance, CD56-positive and perforin-containing EVs from NK cells can mediate EV-induced cytotoxicity [20]. Several immunosuppressive effects of EVs have also been reported [21, 32, 33]; for instance, Fas Ligand (Fas-L)^+^ EVs released from regulatory T cells are able to inhibit DC-induced cytotoxic T-lymphocyte (CTL) responses [21]. Furthermore, inhibitory roles of EVs derived from immature DCs have been observed in relation to transplant tolerance [34–36]. Thus, identification of a specific molecular signature of EVs released by immune cells can provide knowledge that can lead to the use of EVs in a therapeutic setting.

Numerous studies have investigated the effects of EVs released from different leukocytes, leading to an incipient understanding of the physiological functions of these EVs. Nevertheless, several basic questions concerning specific characteristics, like the protein composition of the different types of leukocyte-derived EVs, remain unclear. The present study investigated the expression of selected immunological lineage-specific markers and selected vesicle-related markers on five major leukocyte subpopulations, namely, CD4^+^ T cells, CD8^+^ T cells, NK cells, B cells, and monocytes. The expression was determined for leukocytes present in freshly isolated whole blood and on cultured isolated leukocyte subpopulations and compared to the presentation of these markers on the corresponding leukocyte-derived EVs. The EV Array, used for phenotyping of EVs, is optimized for detection of small EVs with a size below 150 nm that present CD9, CD63, and/or CD81, such as exosomes and exosome-like vesicles. However, as these markers may be present on several types of EVs and as the intracellular origin of the characterized EVs was not determined, the EV subset investigated in the current study was denoted by small EVs (sEVs).

## 2. Materials and Methods

### 2.1. Biological Samples

Blood samples were obtained from ten healthy volunteers at the Department of Clinical Immunology, Aalborg University Hospital. From each donor two blood samples were collected, one tube containing EDTA (K3EDTA, Vacuette®, Greiner Bio-one, Austria) for immediate analysis of noncultured leukocytes and one tube containing CPDA (Vacuette, Greiner Bio-one, Austria) for the vesicle analysis (plasma). Plasma was isolated by centrifugation at 1800 ×g for 6 min at room temperature (RT), after which the plasma supernatant was aliquoted and stored at −40°C until analysis. Buffy coats were obtained from three healthy donors at the Department of Clinical Immunology, Aalborg University Hospital, and used for isolation of peripheral blood mononuclear cells (PBMCs). Blood samples and buffy coats were not obtained from the same donors.

### 2.2. Isolation of Leukocyte Subpopulations

Buffy coats were diluted (1 : 4) in sterile PBS (sPBS) and PBMCs were isolated by density gradient centrifugation using Lymphoprep (Axis-Shield, Oslo, Norway). PBMCs were subsequently washed twice in growth medium (RPMI1640 (Gibco, Life Technologies, Carlsbad, CA, USA), 10% heat-inactivated fetal calf serum (FSC) (Gibco), 100 U/mL penicillin, and 10 *μ*g/mL streptomycin (Amplicon, Odense, Denmark)) and counted in trypan blue and resuspended in isolation buffer (Ca^2+^- and Mg^2+^-free PBS, 2 mM EDTA, and 0.1% bovine serum albumin (BSA)). Magnetic Dynabeads® were used for isolation of human CD4^+^ and CD8^+^ T cells according to the manufacturer's instructions (Dynabeads CD4 Positive Isolation Kit and Dynabeads CD8 Positive Isolation Kit, Life Technologies). Briefly, 1 × 10^7^ cells/mL were mixed with washed beads and incubated for 20 min at 4°C with gentle rotation. The cell suspension was subsequently placed in a magnet for 3-4 min. The supernatant was removed and the bead-bound cells were incubated with Detachabeads® for 45 min at RT with gentle rotation. The cell suspension was placed in a magnet for 3-4 min and the supernatant containing the detached cells was collected. The detached cells were washed once in growth medium and adjusted to 3 × 10^6^ cells/mL. From the CD4^+^ depleted PBMCs, B cells were isolated using the Dynabeads Untouched*™* Human B Cells kit (Life Technologies) according to the manufacturer's instructions. From the CD8^+^ depleted PBMCs, monocytes were isolated using the Dynabeads Untouched Human Monocytes kit (Life Technologies) according to the manufacturer's instructions. Human NK cells were isolated from PBMCs by negative selection according to the manufacturer's instructions (Dynabeads Untouched Human NK Cells kit, Life Technologies). Briefly, 1 × 10^8^ cells/mL were mixed with the antibody cocktail and incubated for 20 min at 4°C followed by washing and incubation with Dynabeads for 15 min at 4°C while rotating. The cell suspension was subsequently placed in a magnet for 3-4 min. The supernatant, containing the cells of interest, was removed and centrifuged at 500 ×g, for 5 min at RT. The pellet was washed once in growth medium and adjusted to 3 × 10^6^ cells/mL. For each isolation, the purity of the cells was determined by flow cytometry.

### 2.3. Culturing of Isolated Subpopulations and Harvest of Cells and sEVs

Isolated leukocytes were adjusted to 3 × 10^6^ cells/mL and cultured in either 12-well (3 × 10^6^ cells/well, 1.5 mL/well) or 24-well plates (1.5 × 10^6^ cells/well, 1 mL/well) (Nunc, Thermo Scientific, Carlsbad, CA, USA) for 44–48 hours at 37°C and 5% CO_2_. Following incubation, the plates were centrifuged at 600 ×g for 10 min at RT and the supernatants containing the cell-derived EVs were harvested from the plates. Complete protease inhibitor, EDTA-free (Roche, DE, USA), was added to the EV-rich supernatants, which were subsequently upconcentrated using Amicon Ultra 100K spin columns according to the manufacturer's instructions. Briefly, PBS was added to the harvested supernatants (total volume of 5 mL) and the supernatants were subsequently centrifuged at 600 ×g for 7 min at RT. The supernatants were transferred to 50 mL spin columns and centrifuged at 3200 ×g for 10 min at RT. Prior to adding the supernatants, the spin columns were washed once in 5 mL PBS (3200 ×g, 10 min, RT). Following the first centrifugation of the supernatant, 5 mL of PBS was added to the retention volume and centrifuged again. This procedure was repeated. The retention volume, containing the upconcentrated supernatants, was harvested and the filter unit was washed in 50–100 *μ*L PBS. Due to varying cell counts between the subpopulations and across the experiments, every supernatant was adjusted to a volume corresponding to 5.5 × 10^6^ cells/mL. No further purification of the EVs was performed and the upconcentrated supernatants were aliquoted and stored at −40°C until analysis by the EV Array. The cells were harvested in PBS, washed, and resuspended in PBS with 0.5% BSA and 0.09% NaN_3_, followed by a subsequent surface marker staining.

### 2.4. Antibodies

For the EV Array, the following antibodies were used for capturing: CD11a (HI111) from Ab Biotech (San Diego, CA, USA); Flotillin-1 and TSG101 (5B7) from Abcam (Cambridge, MA, USA); CD3 (HIT3a), CD14 (M5E2), CD16 (3G8), CD28 (L293), CD49d (L25), and CD56 (3G8) from BD Biosciences (Mountain View, CA, USA); CD41 (HIP8), CD63 (MEM-259), HLA-ABC (W6/32), and Alix (3A9) from Biolegend (San Diego, CA, USA); ICAM-1 (R6.5) from eBiosciences (San Diego, CA, USA); CD9, CD42a, CD81, and CTLA-4 (ANC152.2/8H5) from LifeSpan BioSciences (Seattle, WA, USA); Annexin V (AF399), CD4 (34930), CD8*α* (37006), CD19 (4G7-2E3), CD37 (424925), CD45 (2D1), CD80 (37711), CD82 (423524), CD83 (HB15e), MIC A/B (159207), TNF RI, and TNF RII from R&D Systems (Minneapolis, MN, USA); TLR3 (3.7) from Santa Cruz Biotechnologies (Dallas, TX, USA); HLA-DR/DP/DQ (HB-145) from Loke Diagnostics (Aarhus, Denmark); Fas Ligand (10F2) from Serotec (Oxford, UK); and PD-L1 from Sino Biological (Beijing, China). The following antibodies were used for detection (biotinylated): CD9, CD63, and CD81 from LifeSpan BioSciences.

For flow cytometry the following antibodies were used: CD45 APC (T29/33), CD45 FITC (T29/33), and CD3 FITC (UCHT1) from DakoCytomation (Glostrup, Denmark); CD3 APC (UCHT1), CD4 FITC (RPA-T4), CD4 APC-H7 (SK3), CD8 APC-H7 (SK1), CD9 PerCP-Cy5.5 (M-L13), CD14 FITC (M5E2), CD16 FITC (3G8), CD16 PE-Cy7 (3G8), CD19 APC (HIB19), CD56 APC (B159), and CD56 PE-Cy7 (B159) from BD Biosciences (Mountain View, CA, USA); CD63 PE-Cy7 (H5C6) from eBiosciences; and CD81 PE from LifeSpan BioSciences. In addition, isotype- and fluorophore-matched control antibodies were included.

### 2.5. Flow Cytometry

For analysis of noncultured leukocytes, 100 *μ*L of freshly drawn whole blood was labeled with fluorescence-conjugated antibodies for 30 min at RT, red blood cells were lysed, and the remaining leukocytes were washed and resuspended in BD FACSflow Sheath Fluid (BD Biosciences) with 1% paraformaldehyde.

The cultured leukocytes were labeled with fluorescence-conjugated antibodies for 30 min at RT. The leukocytes were washed twice in PBS with 0.5% BSA and 0.09% NaN_3_ and resuspended in BD FACSflow Sheath Fluid (BD Biosciences) with 1% paraformaldehyde.

Cells were analyzed on a FACSCanto II flow cytometer (BD Biosciences) using the BD FACSDiva*™* Software version 6.1.3 (BD Biosciences). The acquired data files were analyzed by FlowJo vX.0.7 (TreeStar, Ashland, USA), first by adding a leukocyte-gate based on morphologic characteristics (FSC/SSC) and subsequently by the use of the lineage-specific markers; CD3 and CD4 for CD4^+^ T cells; CD3 and CD8 for CD8^+^ T cells; CD16 and CD56 for NK cells; CD19 for B cells; and CD14 for monocytes.

### 2.6. EV Array

For the production of the protein microarray, microarray printing was performed on a SpotBot® Extreme protein edition microarray printer with a 946MP4 pin (ArrayIt, Sunnyvale, CA, USA). Epoxy coated slides (75.6 mm × 25.0 mm; SCHOTT Nexterion, Jena, Germany) were used as microarray basis. The antibodies listed above were diluted in PBS containing 5% glycerol and printed at a concentration of 180–200 *μ*g/mL. As a positive control, 100 *μ*g/mL of biotinylated human IgG in PBS with 5% glycerol was printed. As a negative control, PBS with 5% glycerol was printed.

For catching, visualization, and data analysis, the procedures were performed as previously described [37]. In short, the slides were incubated with 100 *μ*L plasma, prediluted 1 : 10, or 100 *μ*L upconcentrated cell culture supernatant, prediluted 1 : 2 in wash buffer (PBS with 0.05% Tween-20®). All samples were analyzed in triplicate. After overnight sample incubation and a subsequent wash, the slides were incubated with a cocktail of biotinylated detection antibodies (anti-human CD9, CD63, and CD81), diluted 1 : 1500 in wash buffer. After incubation, Cy5-labeled streptavidin (Invitrogen, Frederick, MD, USA) diluted 1 : 1500 was used for detection. Prior to scanning, the slides were washed and dried using a Microarray High-Speed Centrifuge (ArrayIt). Scanning and data analysis was performed as previously described [37]. Briefly, the intensity of the antibody signal was calculated by subtracting the mean of the background (without sample/blank) from the mean of the triplicate antibody spots. This signal was then divided by the signal from the mean of the triplicate negative spots (without capture antibody, with sample). This relative fluorescence intensity was subsequently log⁡2 transformed.

## 3. Results

Leukocytes are immune cells responsible for recognizing and eliminating invading pathogens. Leukocytes can be subdivided based on morphological characteristics upon staining into polymorphonuclear cells (the granulocytes) and mononuclear cells (the lymphocytes and the monocytes). The present study investigated the sEVs produced by the different subpopulations of mononuclear cells found in peripheral blood and compared the vesicular phenotype to the cellular phenotype. In addition, sEVs present in plasma were phenotyped for the same panel of markers.

### 3.1. Phenotypic Characterization of sEVs

**Figure 1:**
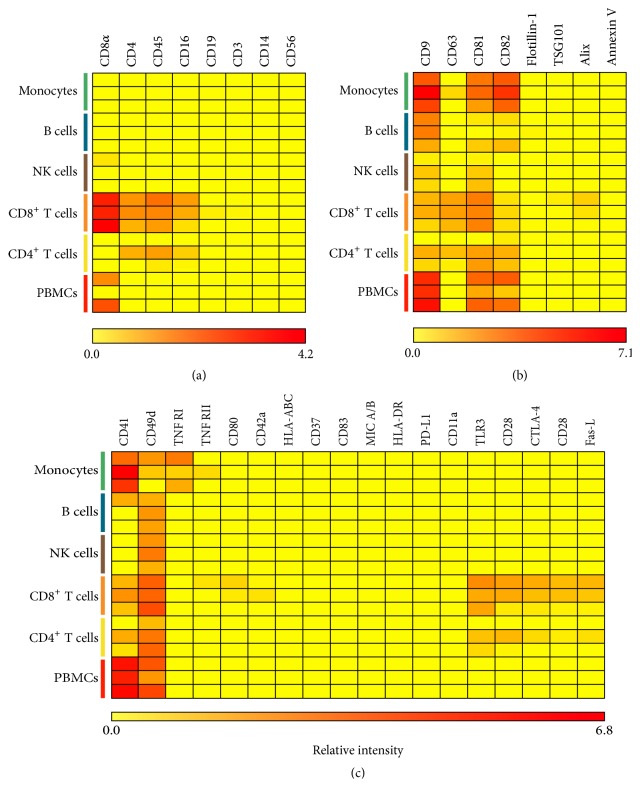
Phenotyping of the different subsets of leukocyte-derived sEVs. The different subsets of leukocyte-derived sEVs were phenotyped for a panel of selected markers using the EV Array. The sEVs were captured by antibodies targeting the selected markers and subsequently detected by addition of an antibody cocktail against CD9, CD63, and CD81. The heat maps illustrate the results from each of the three independent experiments divided into lineage-specific markers (a), vesicle-related markers (b), and general immunological markers (c) and present the relative intensities of each of the markers as indicated by the colored bars. Data are presented as the mean value of the triplicates.

Five different subpopulations of leukocytes were isolated from PBMCs. The purity of the different subpopulations throughout the three individual experiments was 97–99.5% for the CD4^+^ and the CD8^+^ T cells, 82–95% for the B cells, 68–94% for the monocytes, and 85–97% for the NK cells. The isolated subpopulations of leukocytes were cultured for 44–48 hours and the supernatants, containing the leukocyte-produced EVs, were investigated for the presence of a panel of selected immunological and EV-related markers using the EV Array (Table 1). It was investigated whether sEVs from isolated leukocyte subpopulations presented the lineage-specific markers that define the parent cells. The results demonstrated that not all lineage-specific markers could be observed on the corresponding sEV subpopulations (Figure 1(a)). For instance, CD19, which is a well-defined lineage marker for B cells, could not be detected on sEVs in supernatant from cultured B cells. Likewise, CD14, CD16, CD56, and CD3 could not be detected on sEVs from cultured monocytes, NK cells, and T cells, respectively (Figure 1(a)). In contrast, CD4 and CD8, which are lineage markers for two different populations of T cells, were detected on sEVs from the supernatants of these two T cell subpopulations. In addition, sEVs from cultured PBMCs presented CD8, but none of the other markers. Aside from the parental lineage-specific markers, the presentation of other lineage-specific markers was investigated. Small EVs released from CD8^+^ T cells presented CD4, CD45, and CD16. Likewise, CD45 and CD16 could be detected in one of the experiments with sEVs from CD4^+^ T cells.

A panel of EV-related markers was assayed on the different subsets of the leukocyte-derived EVs (Figure 1(b)). CD9 was detected on all sEV subsets, though at very low levels on sEVs from T cells and NK cells. Similarly, CD81 was detected on all sEVs but at very low levels on sEVs from B cells and NK cells. CD82 was detected at high levels on sEVs from monocytes and PBMCs and at very low levels on sEVs from B cells and T cells but was absent on sEVs from NK cells. CD63 was only detected on sEVs from cultured T cells. Furthermore, Alix was detected at very low levels on sEVs from the cultured CD8^+^ T cells. In contrast, Annexin V, TSG101, and Flotillin-1 could not be detected on the leukocyte-derived sEVs.

**Figure 2:**
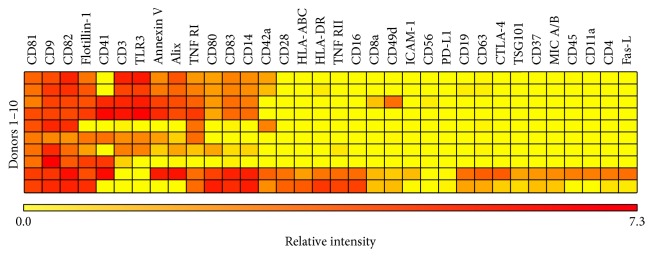
Phenotyping of sEVs present in plasma. Extracellular vesicles present in plasma from 10 healthy donors were investigated for 34 different immune-related or vesicle-related markers using the EV Array. The sEVs were captured by the selected markers and subsequently detected by addition of an antibody cocktail against CD9, CD63, and CD81. The heat map presents the relative intensities for each of the markers as indicated by the colored bar. Data are presented as the mean value of the triplicates from each of the ten donors.

Furthermore, the presence of more general immunological markers was investigated on the different sEV populations. A total of 18 markers were chosen based on their relevance for the function of leukocytes (Table 1). The majority of markers could not be detected on the sEV subpopulations using the EV Array (Figure 1(c)). However, CD49d was found on sEVs from all the different leukocyte subpopulations and CD41 was detected on sEVs from all subpopulations besides sEVs from NK cells. Additionally, sEVs from cultured CD4^+^ and CD8^+^ T cells presented several of the immunological markers, including CD11a, TLR3, CD28, CTLA-4, and the Fas-L. TNF RI was only observed on sEVs from cultured monocytes. The data presented in the heat maps were from three individual experiments and illustrated a degree of variation between the individuals. The presence of a natural variation in the phenotype of sEVs between healthy individuals was also observed upon phenotyping of the sEVs present in plasma from 10 healthy individuals (Figure 2). Plasma sEVs presented several of the markers that were detected on the leukocyte-derived sEVs. In addition, plasma sEVs presented some lineage-specific markers, including CD3, CD14, and CD19 that were not detected on sEVs from cultured leukocytes. Furthermore, TNF RI, TLR3, CD42a, CD80, and CD83 as well as Annexin V, Flotillin-1, and Alix were detected on the majority of the plasma sEVs.

### 3.2. The Cellular Expression Level of CD9, CD63, and CD81 on Freshly Isolated or Cultured Leukocytes

**Figure 3:**
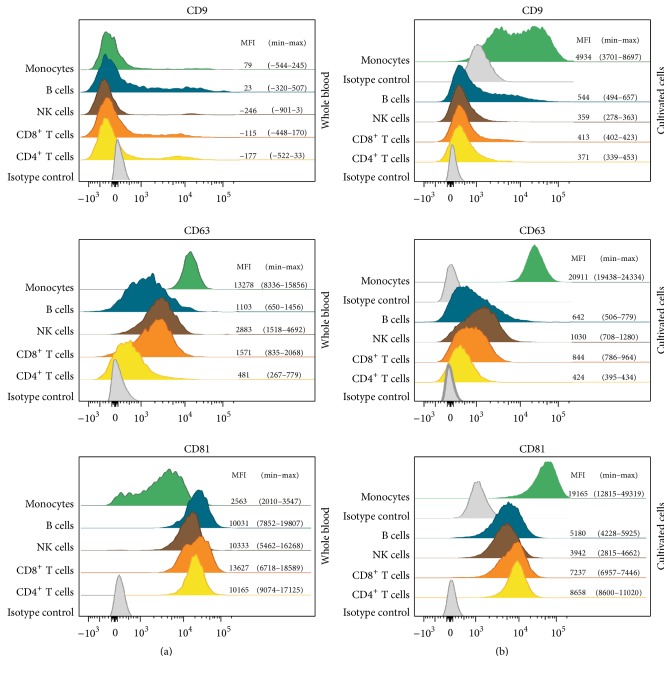
Cellular expression of CD9, CD63, and CD81 on five leukocyte subpopulations. The cellular expression pattern of CD9, CD63, and CD81 was investigated for leukocytes present in freshly drawn whole blood (a) as well as for isolated subpopulations of leukocytes following two days of culture (b). For each of the subpopulations, the expression pattern was determined by flow cytometry. The expression from two representative donors is illustrated in histograms. Isotype controls were included for every leukocyte subpopulation and when the obtained signals were similar, only one isotype control is depicted. Regarding the cultivated cells, a representative lymphocyte isotype control is shown. Due to differences in the level of the isotype control between the lymphocytes and the monocytes, the monocyte isotype control is also shown. The interdonor variation in the expression levels of the different markers is displayed next to each histogram. Data are presented as median fluorescence intensities (MFI) with min and max indicated in parenthesis from the ten (whole blood) or three donors (cultivated cells), respectively.

The cellular expression pattern of CD9, CD63, and CD81 was determined for all the leukocyte subpopulations by flow cytometry (Figure 3). The expression of these vesicle-related markers was investigated for leukocytes present in freshly drawn whole blood (*n* = 10) as well as for isolated subpopulations of leukocytes following two days of culture (*n* = 3). Figures 3(a) and 3(b) demonstrate the expression levels in histograms from two representative donors. None of the leukocyte subsets from whole blood presented CD9 on their surface (Figure 3(a)). In contrast, both CD63 and CD81 were expressed on all leukocytes, but the expression pattern varied between the different subsets. The lymphocytes, including the CD4^+^ T cells, the CD8^+^ T cells, the B cells, and the NK cells, expressed CD81 at high levels, while they all expressed CD63 at low-to-intermediate levels. The opposite situation was observed for the monocytes that presented high levels of CD63 on their surface, but only low-to-intermediate levels of CD81. Regarding the cultured leukocytes, each subpopulation was isolated from three different donors and cultured for 44–48 hours (Figure 3(b)). The results showed that, in contrast to the freshly isolated leukocytes, the cultured leukocytes presented CD9 on their surface. For the lymphocyte populations, the expression of CD9 was low, while the monocyte population expressed intermediate levels of CD9. All cultured lymphocyte subpopulations expressed CD63 and CD81 at low-to-intermediate levels, while the monocytes expressed high levels of both markers. Even though the levels varied, the expression patterns of CD63 and CD81 on the cultured leukocyte subpopulations were similar to the patterns observed on leukocytes present in freshly isolated blood. Based on the obtained minimum and maximum MFI values, it was clear that the expression level of the three markers varied between the individuals illustrating a natural variation.

### 3.3. Cellular versus Vesicular Presentation of Markers

**Figure 4:**
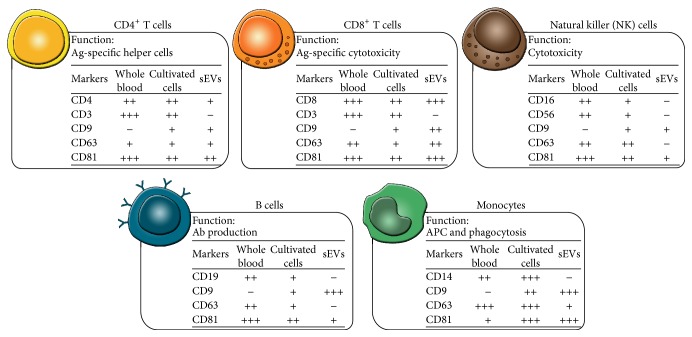
Summary of results on the presence of selected lineage-specific and vesicle-related markers on five leukocyte subpopulations and the corresponding leukocyte-derived sEVs. The expression of selected lineage-specific and vesicle-related markers was determined for freshly isolated leukocytes and for cultured leukocyte subpopulations. The cellular expression was determined by flow cytometry. The level of the different markers was grouped into negative (−), low (+), intermediate (++), or high (+++) corresponding to the following signal intensities: <100, 100–1000, 1000–10000, and 10000–100000, respectively. The results presented on freshly isolated leukocytes and on cultured leukocytes are based on median values of ten or three individual experiments, respectively. The EV Array determined the presence of the markers on the corresponding leukocyte-derived sEVs. The level of the different markers is grouped into negative (−), low (+), intermediate (++), or high (+++) corresponding to the following signal intensities: 0–0.1, 0.1–1, 1–2.5, and 2.5–5.5, respectively. The results presented on the leukocyte-derived sEVs are based on mean values of three individual experiments. Ag: antigen; Ab: antibody; APC: antigen-presenting cell.

In order to investigate the phenotypic homogeneity between the sEVs and the parent cells, the cellular expression patterns of selected lineage-specific and vesicle-related markers were compared to the vesicular presentation of these markers (Figure 4). In relation to the selected lineage-specific markers, CD3, CD14, CD16, and CD19 could not be detected on sEVs from cultured T cells, monocytes, NK cells, and B cells, respectively, indicating that not all cell surface molecules are transferred to the sEV surface. For CD9, CD63, and CD81, the majority of the subpopulations presented vesicular levels that overall correlated with the cellular expression. Exceptions were observed with CD63, in which case the cellular expression on monocytes was high, but the vesicular presentation was low. Similarly, the cellular expression of CD63 was intermediate or low for both NK cells and B cells, but no CD63 could be detected on the corresponding sEVs.

## 4. Discussion

Several studies have reported that EVs are released from different leukocyte subsets; however, a thorough simultaneous investigation of the major leukocyte subsets with focus on lineage-specific markers has not yet been described. In the current study the phenotype of five different subsets of leukocyte-derived sEVs was determined and related to both the phenotype of sEVs present in plasma and the cellular phenotype of the leukocyte subpopulations. The EV Array, used for the investigation of the phenotype of sEVs, is a protein microarray technique that provides the opportunity to detect and characterize sEVs for up to 60 markers in a high-throughput manner and with high sensitivity [37, 38]. One major advantage of the EV Array is the ability to phenotype sEVs from unpurified material without a requirement for preanalytical purification. Isolation of specific EV subsets is in many cases highly warranted, but for phenotyping of EVs using the EV Array, crude plasma or cell-free supernatants are applicable.

In order to investigate whether lineage-specific proteins present on leukocytes could be detected on the corresponding sEVs, leukocytes were phenotyped for selected lineage-specific markers using flow cytometry, while the sEVs were phenotyped using the EV Array. The results showed that none of the included lineage-specific markers could be detected on the sEVs released from B cells, NK cells, and monocytes in monocultures (CD19, CD56, and CD14, resp.). In contrast, sEVs from monocultures of CD4^+^ and CD8^+^ T cells did present CD4 and CD45 or CD8 and CD45, respectively. None of the T cell-derived sEVs presented CD3. A study by Kornek et al. investigated EVs from Jurkat T cells activated with phytohemagglutinin (PHA) and observed that the fraction of large EVs (sedimented at 10.000 ×g) only presented low levels of CD3, while the fraction of sEVs (sedimented at 100.000 ×g) presented high levels of CD3 [39]. Regarding CD19, a study by Admyre and coworkers from 2007 showed that EVs from a B cell line presented CD19 [40], which differs from the observations obtained in the study at hand. Similarly, another study has described that EVs from resting NK cells present typical NK cell markers like CD56 and Fas-L, but not CD16 [20]. In terms of CD14, a study by Aharon et al. observed this marker on large EVs (MVs) released from a monocytic cell line [41]. No CD14 could be detected on sEVs from cultured monocytes in the current study. The discrepancy between some of the results may be due to investigations of different preparations of EVs and/or may be explained by the fact that the EV-producing cells were different, for example, cells isolated from peripheral blood versus cell lines. In addition, differences in the activation state of the EV-producing cells are of importance for the outcome. Upon stimulation, cells change their phenotype and this phenotypic change depends on the specific stimulus given to the cells [28, 42]. In the present study, leukocyte subpopulations were isolated and cultured without any activation signal, while several of the other studies investigated the phenotype of the EVs released upon adding a stimulus to the cells. The presented results obtained with sEVs released from cultured PBMCs support this phenomenon as these sEVs apparently displayed fewer markers than sEVs from monocultures. Overall, these data indicate that the phenotype of the vesicle subset is context-dependent. However, during the current conditions, several of the leukocyte-derived sEVs did not present the lineage-specific markers found on the parent cell type (Figure 1(a)). Variations in the molecular content between cells and EVs were also observed in a study by Hunter et al. that reported significant differences in the presence of miRNAs between plasma EVs and PBMCs [43]. For comparison, the presentation of lineage-specific markers on plasma sEVs was determined. In contrast to the leukocyte-derived sEVs, plasma sEVs presented several lineage-specific markers, including CD3, CD14, and CD19. Compared to the EVs present in cell supernatants, EVs in plasma are a mixture of EVs produced by many different cell types. Thus, plasma can act as a liquid biopsy and will present a very heterogeneous EV phenotype that provides information about numerous cellular processes. The detection of CD3, CD14, and CD19 on plasma sEVs, but not on sEVs from cultured leukocyte subpopulations, emphasizes that the biological context has a great impact on the phenotype of the released EVs.

Interestingly, some of the sEV subsets presented other markers, for example, sEVs from CD8^+^ T cells presenting CD16. CD16 was also detected on plasma sEVs from some of the healthy donors. Several studies have described the expression of CD16 on a small portion of different types of T cells and even shown a functional role of CD16 on T cells [44, 45]. Thus, the observations of CD16 on sEVs released from cultured T cells combined with the fact that functional molecules, such as perforin and lytic granules, can be stored inside EVs may indicate a specific functionality of these EVs, for example, surrogates or extensions of the effector cell in antibody-dependent, cell-mediated cytotoxicity.

Apart from the selected lineage-specific markers, the sEVs were phenotyped for the presence of 26 different immunological or vesicle-related markers. Regarding the leukocyte-derived sEVs, CD49d was the only marker detected on all sEV subsets (Figure 1(c)). CD49d is an *α*_4_ integrin that is expressed as a heterodimer on T, B, and NK cells and monocytes as well as on other immune cells. Together with CD29, CD49d forms the very late antigen-4 (VLA-4) that is involved in leukocyte trafficking, adhesion, and extravasation [46, 47]. So far, two studies have reported on the presence of CD49d/VLA-4 on EVs from B cells [48, 49], and these results can be confirmed by the present study. In terms of plasma sEVs, CD49d was detected in three of the ten healthy donors. Similar result was obtained in a study by Peinado et al., demonstrating weak VLA-4 expression on plasma EVs from healthy donors [50]. Surprisingly, CD41 was observed on sEVs from T cells, B cells, monocytes, and PBMCs as well as on plasma sEVs from half of the healthy donors (Figure 1(c)). CD41 is a transmembrane glycoprotein primarily expressed on platelets and megakaryocytes. However, according to the Protein Atlas, CD41 is expressed by PBMCs [51] and even though the function is unclear, the presence of CD41 on sEVs released from these cells, either in monocultures or as PBMCs, is possible. In addition, sEVs from cultured T cells presented an array of other immunological markers, ranging from more general markers, like CD45, to specific markers like CD28, and the TLR3. Moreover, sEVs from CD8^+^ T cells presented CTLA-4 and the Fas-L. Even though the mechanisms are quite different, both of these molecules play a role in downregulation of an immune response, either by transmitting an inhibitory signal in activated T cells or by inducing activation-induced cell death, respectively. The presence of such regulatory molecules on EVs suggests that T cell-derived EVs can be involved in immune regulation. The presence of Fas-L on EVs from T cells has also been observed in other studies [52–54], but to the best of our knowledge, studies observing CTLA-4 on EVs from leukocytes have not previously been published. Upon evaluating the presence of markers on EVs, it is important to consider the natural variation in the molecular composition of the EV pool that exists between healthy individuals as visualized by the ten plasma samples. Thus, the present results are a snapshot providing insight about the current vesicular presentation of selected markers.

The sEVs investigated in this study were identified by the presence of CD9, CD63, and/or CD81. Thus, in order to investigate the similarity of the leukocytes and the corresponding sEVs, the cellular expression patterns of CD9, CD63, and CD81 were determined. The expression pattern was determined for both leukocytes present in whole blood and cultured leukocyte subpopulations. The results demonstrated a lack of CD9 on the surface of leukocytes present in freshly drawn whole blood (Figure 3(a)). In contrast, CD9 was present on every leukocyte subpopulation following two days of culture. In accordance with the present results, a recently published study also observed CD9 on the surface of isolated populations of CD3^+^, CD14^+^, and CD19^+^ cells [55]. The expression patterns of CD63 and CD81 were quite similar between the two cell preparations but with minor differences in the expressions levels. Overall, these findings are in agreement with previously described results [56–61]. When looking at both the cellular and the vesicular presentation of these markers, it is clear that, for the majority of the cultured subpopulations, the presentation of these markers was comparable. However, for the NK cells, the B cells, and the monocytes, the cellular expression of CD63 was very different from the presentation observed on sEVs. A comparison between the presentation of CD9, CD63, and CD81 on whole blood leukocytes and plasma EVs is a more complex procedure that needs to take into account that plasma EVs are a very heterogeneous group of EVs that emanate from multiple cell types. The tetraspanin signals observed on the plasma sEVs represent all tetraspanin-positive sEVs irrespective of their origin. Thus, the presentation of tetraspanins on plasma sEVs cannot be expected to correlate with the expression of tetraspanin on leukocytes. Nevertheless, it is clear that the CD63 signals in plasma generally were low, while the cellular expression of CD63 for several of the leukocyte subpopulations is medium to high, indicating that the presentation of CD63 is different from sEVs to cells. Overall, the vesicular signal intensities for CD63 were much lower than the intensities observed for CD9 and CD81, suggesting that CD63 might be a poor marker for sEVs in general, which has also been observed in other studies [38, 62–64]. The results underline the importance of detecting EVs with a cocktail of antibodies against tetraspanins, as detection with a single marker may overlook some subsets of EVs.

## 5. Conclusion

Surface molecules on EVs are responsible for the biodistribution and the ligation to target cells. Thus, the protein composition is related to the functionality of EVs, why phenotyping of EVs can be used to gain knowledge about the functionality. In summary, the presented data regarding the lineage-specific markers and the tetraspanins support the accumulating observations suggesting that the transfer of molecular cargo into EVs is tightly controlled. A tightly regulated sampling of molecules to EVs would match with the fact that EVs play an important role as systemic regulators, traveling through tissues providing key intercellular communication as well as transfer of biologically active components.

## Figures and Tables

**Table 1:** Overview of the markers selected for phenotyping of sEVs.

Immunological markers				Vesicle-related markers	
Functional	Regulatory	Adhesion	Lineage-specific and others	Tetraspanins	Others

HLA-DR	CTLA-4	CD11a	CD3	CD9	TSG101
HLA-ABC	PD-L1	ICAM-1	CD4	CD63	Alix
CD28			CD8	CD81	Flotillin-1
CD80			CD14	CD82	Annexin V
CD49d			CD16		
TLR3			CD19		
Fas-L			CD37		
MIC A/B			CD41		
TNF RI			CD42a		
TNF RII			CD45		
			CD56		
			CD83		

HLA: human leukocyte antigen; TLR: Toll-like receptor; TNF: tumor necrosis factor: RI: receptor I, RII: receptor II; MIC A/B: major histocompatibility complex class I-related chain A/B; CTLA-4: cytotoxic T-lymphocyte associated protein-4; PD-L1: programmed death-ligand 1; ICAM-1: intercellular adhesion molecule 1; TSG101: tumor susceptibility gene 101.

## Abbreviations

APC: Antigen-presenting cell
CTL: Cytotoxic T-lymphocyte
CTLA-4: Cytotoxic T-lymphocyte associated protein-4
DCs: Dendritic cells
EVs: Extracellular vesicles
Fas-L: Fas Ligand
ICAM-1: Intercellular adhesion molecule 1
MFI: Median fluorescence intensity
MHC-II: Major histocompatibility complex class II
MIC A/B: Major histocompatibility complex class I-related chain A/B
MVs: Microvesicles
NK: Natural killer
NKG2D: Natural killer group 2, member D
PBMCs: Peripheral blood mononuclear cells
PHA: Phytohemagglutinin
sEVs: Small EVs
TLR: Toll-like receptor
TNF RI: Tumor necrosis factor receptor-1
TSG101: Tumor susceptibility gene 101
VLA-4: Very late antigen-4.

## References

[1] Revenfeld A. L. S., Bæk R., Nielsen M. H., Stensballe A., Varming K., Jørgensen M. (2014). Diagnostic and prognostic potential of extracellular vesicles in peripheral blood. *Clinical Therapeutics*.

[2] Yáñez-Mó M., Siljander P. R.-M., Andreu Z. (2015). Biological properties of extracellular vesicles and their physiological functions. *Journal of Extracellular Vesicles*.

[3] Théry C., Zitvogel L., Amigorena S. (2002). Exosomes: composition, biogenesis and function. *Nature Reviews Immunology*.

[4] Gould S. J., Raposo G. (2013). As we wait: coping with an imperfect nomenclature for extracellular vesicles. *Journal of Extracellular Vesicles*.

[5] Valadi H., Ekström K., Bossios A., Sjöstrand M., Lee J. J., Lötvall J. O. (2007). Exosome-mediated transfer of mRNAs and microRNAs is a novel mechanism of genetic exchange between cells. *Nature Cell Biology*.

[6] Denzer K., Kleijmeer M. J., Heijnen H. F. G., Stoorvogel W., Geuze H. J. (2000). Exosome: from internal vesicle of the multivesicular body to intercellular signaling device. *Journal of Cell Science*.

[7] Théry C., Ostrowski M., Segura E. (2009). Membrane vesicles as conveyors of immune responses. *Nature Reviews Immunology*.

[8] Huber V., Filipazzi P., Iero M., Fais S., Rivoltini L. (2008). More insights into the immunosuppressive potential of tumor exosomes. *Journal of Translational Medicine*.

[9] Iero M., Valenti R., Huber V. (2008). Tumour-released exosomes and their implications in cancer immunity. *Cell Death and Differentiation*.

[10] Oksvold M. P., Kullmann A., Forfang L. (2014). Expression of B-Cell surface antigens in subpopulations of exosomes released from B-cell lymphoma cells. *Clinical Therapeutics*.

[11] Muralidharan-Chari V., Clancy J. W., Sedgwick A., D'Souza-Schorey C. (2010). Microvesicles: mediators of extracellular communication during cancer progression. *Journal of Cell Science*.

[12] Valenti R., Huber V., Filipazzi P. (2006). Human tumor-released microvesicles promote the differentiation of myeloid cells with transforming growth factor-*β*-mediated suppressive activity on T lymphocytes. *Cancer Research*.

[13] Taylor D. D., Gerçel-Taylor C., Lyons K. S., Stanson J., Whiteside T. L. (2003). T-cell apoptosis and suppression of T-cell receptor/CD3-zeta by Fas ligand-containing membrane vesicles shed from ovarian tumors. *Clinical Cancer Research*.

[14] Mack M., Kleinschmidt A., Brühl H. (2000). Transfer of the chemokine receptor CCR5 between cells by membrane-derived microparticles: a mechanism for cellular human immunodeficiency virus 1 infection. *Nature Medicine*.

[15] Wurdinger T., Gatson N. N., Balaj L., Kaur B., Breakefield X. O., Pegtel D. M. (2012). Extracellular vesicles and their convergence with viral pathways. *Advances in Virology*.

[16] Mittelbrunn M., Gutiérrez-Vázquez C., Villarroya-Beltri C. (2011). Unidirectional transfer of microRNA-loaded exosomes from T cells to antigen-presenting cells. *Nature Communications*.

[17] Admyre C., Johansson S. M., Paulie S., Gabrielsson S. (2006). Direct exosome stimulation of peripheral human T cells detected by ELISPOT. *European Journal of Immunology*.

[18] Eldh M., Ekström K., Valadi H. (2010). Exosomes communicate protective messages during oxidative stress; possible role of exosomal shuttle RNA. *PLoS ONE*.

[19] Saunderson S. C., Schuberth P. C., Dunn A. C. (2008). Induction of exosome release in primary B cells stimulated via CD40 and the IL-4 receptor. *The Journal of Immunology*.

[20] Lugini L., Cecchetti S., Huber V. (2012). Immune surveillance properties of human NK cell-derived exosomes. *Journal of Immunology*.

[21] Xie Y., Zhang X., Zhao T., Li W., Xiang J. (2013). Natural CD8^+^25^+^ regulatory T cell-secreted exosomes capable of suppressing cytotoxic T lymphocyte-mediated immunity against B16 melanoma. *Biochemical and Biophysical Research Communications*.

[22] Bhatnagar S., Shinagawa K., Castellino F. J., Schorey J. S. (2007). Exosomes released from macrophages infected with intracellular pathogens stimulate a proinflammatory response in vitro and in vivo. *Blood*.

[23] Nolte-'t Hoen E. N. M., Buschow S. I., Anderton S. M., Stoorvogel W., Wauben M. H. M. (2009). Activated T cells recruit exosomes secreted by dendritic cells via LFA-1. *Blood*.

[24] Garzetti L., Menon R., Finardi A. (2014). Activated macrophages release microvesicles containing polarized M1 or M2 mRNAs. *Journal of Leukocyte Biology*.

[25] Zitvogel L., Regnault A., Lozier A. (1998). Eradication of established murine tumors using a novel cell-free vaccine: dendritic cell-derived exosomes. *Nature Medicine*.

[26] Rialland P., Lankar D., Raposo G., Bonnerot C., Hubert P. (2006). BCR-bound antigen is targeted to exosomes in human follicular lymphoma B-cells. *Biology of the Cell*.

[27] Muntasell A., Berger A. C., Roche P. A. (2007). T cell-induced secretion of MHC class II-peptide complexes on B cell exosomes. *EMBO Journal*.

[28] Segura E., Amigorena S., Théry C. (2005). Mature dendritic cells secrete exosomes with strong ability to induce antigen-specific effector immune responses. *Blood Cells, Molecules, and Diseases*.

[29] Viaud S., Terme M., Flament C. (2009). Dendritic cell-derived exosomes promote natural killer cell activation and proliferation: a role for NKG2D ligands and IL-15R*α*. *PLoS ONE*.

[30] Buschow S. I., Nolte-'t Hoen E. N. M., van Niel G. (2009). MHC II in dendritic cells is targeted to lysosomes or T cell-induced exosomes via distinct multivesicular body pathways. *Traffic*.

[31] Hao S., Yuan J., Xiang J. (2007). Nonspecific CD4^+^ T cells with uptake of antigen-specific dendritic cell-released exosomes stimulate antigen-specific CD8^+^ CTL responses and long-term T cell memory. *Journal of Leukocyte Biology*.

[32] Zhang H., Xie Y., Li W., Chibbar R., Xiong S., Xiang J. (2011). CD4 T cell-released exosomes inhibit CD8 cytotoxic T-lymphocyte responses and antitumor immunity. *Cellular and Molecular Immunology*.

[33] Ashiru O., Boutet P., Fernández-Messina L. (2010). Natural killer cell cytotoxicity is suppressed by exposure to the human NKG2D ligand MICA^∗^008 that is shed by tumor cells in exosomes. *Cancer Research*.

[34] Li X., Li J.-J., Yang J.-Y. (2012). Tolerance induction by exosomes from immature dendritic cells and rapamycin in a mouse cardiac allograft model. *PLoS ONE*.

[35] Yang X., Meng S., Jiang H., Zhu C., Wu W. (2011). Exosomes derived from immature bone marrow dendritic cells induce tolerogenicity of intestinal transplantation in rats. *Journal of Surgical Research*.

[36] Pêche H., Heslan M., Usal C., Amigorena S., Cuturi M. C. (2003). Presentation of donor major histocompatibility complex antigens by bone marrow dendritic cell-derived exosomes modulates allograft rejection. *Transplantation*.

[37] Jørgensen M., Bæk R., Pedersen S., Søndergaard E. K., Kristensen S. R., Varming K. (2013). Extracellular Vesicle (EV) Array: microarray capturing of exosomes and other extracellular vesicles for multiplexed phenotyping. *Journal of Extracellular Vesicles*.

[38] Jørgensen M. M., Bæk R., Varming K. (2015). Potentials and capabilities of the Extracellular Vesicle (EV) Array. *Journal of Extracellular Vesicles*.

[39] Kornek M., Popov Y., Libermann T. A., Afdhal N. H., Schuppan D. (2011). Human T cell microparticles circulate in blood of hepatitis patients and induce fibrolytic activation of hepatic stellate cells. *Hepatology*.

[40] Admyre C., Bohle B., Johansson S. M. (2007). B cell-derived exosomes can present allergen peptides and activate allergen-specific T cells to proliferate and produce TH2-like cytokines. *Journal of Allergy and Clinical Immunology*.

[41] Aharon A., Tamari T., Brenner B. (2008). Monocyte-derived microparticles and exosomes induce procoagulant and apoptotic effects on endothelial cells. *Thrombosis and Haemostasis*.

[42] van der Vlist E., Arkesteijn G. J. A., van de Lest C. H. A., Stoorvogel W., Nolte-'t Hoen E. N. M., Wauben M. H. M. (2012). CD4^+^ T cell activation promotes the differential release of distinct populations of nanosized vesicles. *Journal of Extracellular Vesicles*.

[43] Hunter M. P., Ismail N., Zhang X. (2008). Detection of microRNA expression in human peripheral blood microvesicles. *PLoS ONE*.

[44] Clémenceau B., Vivien R., Debeaupuis E. (2011). Fc*γ*RIIIa (CD16) induction on human t lymphocytes and CD16pos T-lymphocyte amplification. *Journal of Immunotherapy*.

[45] Björkström N. K., Gonzalez V. D., Malmberg K.-J. (2008). Elevated numbers of Fc gamma RIIIA+ (CD16+) effector CD8 T cells with NK cell-like function in chronic hepatitis C virus infection. *The Journal of Immunology*.

[46] Rose D. M., Alon R., Ginsberg M. H. (2007). Integrin modulation and signaling in leukocyte adhesion and migration. *Immunological Reviews*.

[47] Yusuf-Makagiansar H., Anderson M. E., Yakovleva T. V., Murray J. S., Siahaan T. J. (2002). Inhibition of LFA-1/ICAM-1 and VLA-4/VCAM-1 as a therapeutic approach to inflammation and autoimmune diseases. *Medicinal Research Reviews*.

[48] Clayton A., Turkes A., Dewitt S., Steadman R., Mason M. D., Hallett M. B. (2004). Adhesion and signaling by B cell-derived exosomes: the role of integrins. *The FASEB Journal*.

[49] Wubbolts R., Leckie R. S., Veenhuizen P. T. M. (2003). Proteomic and biochemical analyses of human B cell-derived exosomes: potential implications for their function and multivesicular body formation. *The Journal of Biological Chemistry*.

[50] Peinado H., Alečković M., Lavotshkin S. (2012). Melanoma exosomes educate bone marrow progenitor cells toward a pro-metastatic phenotype through MET. *Nature Medicine*.

[51] Uhlen M., Oksvold P., Fagerberg L. (2010). Towards a knowledge-based Human Protein Atlas. *Nature Biotechnology*.

[52] Martínez-Lorenzo M. J., Anel A., Gamen S. (1999). Activated human T cells release bioactive Fas ligand and APO2 ligand in microvesicles. *Journal of Immunology*.

[53] Monleón I., Martínez-Lorenzo M. J., Monteagudo L. (2001). Differential secretion of Fas ligand- or APO2 ligand/TNF-related apoptosis-inducing ligand-carrying microvesicles during activation-induced death of human T cells. *Journal of Immunology*.

[54] Alonso R., Mazzeo C., Rodriguez M. C. (2011). Diacylglycerol kinase *α* regulates the formation and polarisation of mature multivesicular bodies involved in the secretion of Fas ligand-containing exosomes in T lymphocytes. *Cell Death and Differentiation*.

[55] Tsukamoto S., Takeuchi M., Kawaguchi T. (2014). Tetraspanin CD9 modulates ADAM17-mediated shedding of LR11 in leukocytes. *Experimental and Molecular Medicine*.

[56] Tarrant J. M., Robb L., van Spriel A. B., Wright M. D. (2003). Tetraspanins: molecular organisers of the leukocyte surface. *Trends in Immunology*.

[57] Rocha-Perugini V., Zamai M., González-Granado J. M. (2013). CD81 controls sustained T cell activation signaling and defines the maturation stages of cognate immunological synapses. *Molecular and Cellular Biology*.

[58] Todd S. C., Lipps S. G., Crisa L., Salomon D. R., Tsoukas C. D. (1996). CD81 expressed on human thymocytes mediates integrin activation and interleukin 2-dependent proliferation. *The Journal of Experimental Medicine*.

[59] Maecker H. T., Do M.-S., Levy S. (1998). CD81 on B cells promotes interleukin 4 secretion and antibody production during T helper type 2 immune responses. *Proceedings of the National Academy of Sciences of the United States of America*.

[60] Gilsanz A., Sánchez-Martín L., Gutiérrez-López M. D. (2013). ALCAM/CD166 adhesive function is regulated by the tetraspanin CD9. *Cellular and Molecular Life Sciences*.

[61] Tippett E., Cameron P. U., Marsh M., Crowe S. M. (2013). Characterization of tetraspanins CD9, CD53, CD63, and CD81 in monocytes and macrophages in HIV-1 infection. *Journal of Leukocyte Biology*.

[62] Schacht Revenfeld A. L., Lindersson Søndergaard E. K., Stensballe A., Baek R., Møller Jørgensen M., Varming K. (2016). Characterization of a cell-culturing system for the study of contact-independent extracellular vesicle communication. *Journal of Circulating Biomarkers*.

[63] He M., Crow J., Roth M., Zeng Y., Godwin A. K. (2014). Integrated immunoisolation and protein analysis of circulating exosomes using microfluidic technology. *Lab on a Chip—Miniaturisation for Chemistry and Biology*.

[64] Kowal J., Arras G., Colombo M. (2016). Proteomic comparison defines novel markers to characterize heterogeneous populations of extracellular vesicle subtypes. *Proceedings of the National Academy of Sciences*.

